# Defining Age-Adjusted PI–LL Targets for Surgical Realignment in Adult Degenerative Scoliosis: A Retrospective Cohort Study

**DOI:** 10.3390/jcm13133643

**Published:** 2024-06-21

**Authors:** Haoran Zhang, Yuanpeng Zhu, Xiangjie Yin, Dihan Sun, Shengru Wang, Jianguo Zhang

**Affiliations:** Department of Orthopedic Surgery, Peking Union Medical College Hospital, Peking Union Medical College and Chinese Academy of Medical Sciences, Beijing 100730, China; zhanghaoran96@126.com (H.Z.); zhuyuanpeng2022@163.com (Y.Z.); yinxiangjieseu@163.com (X.Y.); sundihan1998@126.com (D.S.)

**Keywords:** adult degenerative scoliosis, corrective surgery, age, PI–LL, spinal deformity, lumbar lordosis

## Abstract

**Objectives:** The purpose of this study was to investigate postoperative pelvic incidence minus lumbar lordosis mismatch (PI–LL) and health-related quality of life (HRQOL) outcomes to determine age-adjusted PI–LL targets. **Method:** The dataset encompassed a range of variables, including age, sex, body mass index, Charlson comorbidity index, presence of osteopenia, hospital stay, operative duration, blood loss, American Society of Anesthesiologists score, number of fusion levels, lumbar lordosis, sagittal vertical axis, pelvic incidence, and PI–LL. The non-linear relationship between PI–LL and clinical outcomes was examined using a curve analysis, with adjustments made for potential confounding variables. Upon identification of a non-linear relationship, a two-piecewise regression model was employed to determine the threshold effect. **Results:** A total of 280 patients were enrolled. In the fully adjusted model, the optimal PI–LL target for patients aged 45–54 years old was PI–LL < 10°, the optimal target for patients aged 55–74 was 10–20°, and the optimal target for patients older than 75 years was more suitable for PI–LL > 20°. In the curve-fitting graph, it could be seen that the relationship between PI–LL and HRQOL outcomes was not linear in each age group. The peaks of the curves within each group occurred at different locations. Higher and lower thresholds for optimal surgical goals were determined using the two-piecewise regression model from the SRS-22 score and the ODI score. **Conclusions:** This study showed that the optimal PI–LL after corrective surgery in adult degenerative scoliosis patients should be adjusted according to age.

## 1. Introduction

The deterioration of standing spinal balance is associated with a reduction in health-related quality of life (HRQOL), with sagittal imbalances contributing most to the decline in HRQOL [1,2,3,4]. Optimal surgical correction of adult degenerative scoliosis (ADS) is critical to improve clinical outcomes and prevent sagittal imbalance. The usual criteria for assessing alignment and planning corrective surgery in adult spinal deformity (ASD) were proposed by Schwab et al. [5]. The suggested reference ranges for pelvic incidence minus lumbar lordosis mismatch (PI–LL) in this classification are fixed [5]. Although these criteria provide a guideline for preoperative planning in most patients with ASD, including adult idiopathic scoliosis and ADS, they appear to be limited in the elderly population [6]. With the gradual development of personalized medicine and precision medicine, one of the goals of modern spinal corrective surgery is to develop a “tailor-made” model to suit an individual’s specific characteristics.

Aging is a degenerative process associated with muscle wasting and neurodegeneration. Aging affects the size, cross-sectional area, and type of muscle fiber, as demonstrated by imaging, physiology, and histopathology [7,8,9,10]. In addition, the thickness of the calcified zone decreased with age, but the number of tidemarks increased, particularly over the age of 60 [11]. Finally, sensory function and postural control function are impaired with aging and may include progressive visual, vestibular, and somatosensory deficits [12]. Therefore, an optimal PI–LL should take these age-related dynamics into account. Mac-Thiong evaluated the HRQOL of 73 adults presenting with scoliosis, and the results showed a significant positive correlation between age and Oswestry Disability Index (ODI) score [13]. Similarly, Baldus et al. prospectively released the HRQOL questionnaire to 1346 adult volunteers recruited from across the United States and found that the older the age-gender group, the lower the reported domain median and mean scores [14].

Some scholars have tried to add age-related parameters to the spinopelvic alignment thresholds. Lafage et al. used regression models to generate radiographic parameters. These values exhibited an age-dependent increase, highlighting the progression of spinal alignment changes and degenerative spinal pathologies as age-related phenomena [15]. However, the population was heterogeneous, and the inclusion criterion was an age greater than 18 years, which resulted in a relatively young population. This led to the fact that the conclusions in this article may not be applicable to elderly patients undergoing corrective surgery for ADS.

Hasegawa et al. clarified the values of spinal alignment changes and balance in healthy Japanese adult volunteers. The results showed that PI–LL was correlated with age, suggesting that spinopelvic harmony deteriorates with age [16]. Similarly, Xu et al. identified independent variables related to lumbar lordosis (LL) through a prospective cross-sectional study and established a prediction formula for ideal LL [17]. Although normative alignment in healthy individuals has been well-documented in this literature, this does not mean that every patient with ADS must be realigned exactly according to the norms of healthy individuals. ADS is a degenerative disease, and corrective surgery should be seen as an intervention for disability, not an outright cure [15]. The current criteria do not add age-related parameters to the spinopelvic alignment thresholds, which may lead to bias in the criteria. The aim was to investigate postoperative PI–LL and HRQOL outcomes to determine age-adjusted PI–LL targets for adult degenerative scoliosis.

## 2. Methods

### 2.1. Study Design

We retrospectively analyzed patients who underwent surgery from January 2011 to June 2021. Inclusion criteria were age ≥ 45 years, coronal Cobb angle of lumbar curves ≥ 20°, posterior internal fixation and fusion ≥ 3 levels, follow-up ≥ 2 years, and complete radiographic measurements and clinical outcomes. We excluded any patients who had a prior diagnosis of scoliosis, had less than 2 years of follow-up, or had indeterminate relevant clinical outcome measurements. Finally, a total of 280 patients with ADS met the criteria and were included in this study. This study was conducted in accordance with the Declaration of Helsinki and was approved by the Institutional Review Board. Informed consents were obtained from all participants.

### 2.2. Data Collection

The data included age, sex, body mass index (BMI), age-adjusted Charlson comorbidity index (aCCI), osteopenia, hospital stay, operative duration, estimated blood loss, American Society of Anesthesiologists (ASA) score, and the number of fusion levels.

Radiographic measurements were obtained preoperatively, immediately postoperatively (1-week postoperatively), and at last follow-up.

Radiographic measurements were LL, sagittal vertical axis (SVA), PI, and PI–LL. PI–LL was calculated using the following formula: PI minus T12-S1 lordosis. Identified patients were stratified into 4 categories: Group A (between 45 and 54 years old), Group B (between 55 and 64 years old), Group C (between 65 and 74 years old), and Group D (age greater than 75 years). Within each cohort, patients were categorized into 3 categories: 0, +, and ++ for PI–LL < 10°, between 10° and 20°, and >20°, respectively.

### 2.3. Clinical Outcomes

HRQOL outcome assessment tools included the Scoliosis Research Society-22 (SRS-22) score and the ODI score [18,19]. An SRS-22 score ≥ 4 or an ODI score ≤ 20 was defined as a favorable clinical outcome [20,21].

### 2.4. Statistical Analysis

The statistical association between PI–LL and clinical outcomes was assessed using logistic regression models. Following the Strengthening the Reporting of Observational Studies in Epidemiology statement [22], we presented results from the crude model, minimally adjusted model, and fully adjusted model. Covariate selection for the multivariate model adhered to three criteria. First, a covariate was included if its addition or removal from the model resulted in a change greater than 10% in the regression coefficient of the independent variable [23]. Second, variables with *p* < 0.1 in univariate analysis were incorporated. Finally, covariates were selected based on relevant literature and clinical expertise from our institution.

Subsequently, we examined the non-linear relationship between PI–LL and clinical outcomes using a smoothed curve analysis, adjusting for potential confounders. In the presence of a non-linear relationship, a two-piecewise regression model was employed to determine the threshold effect of PI–LL on clinical outcomes as indicated by the smoothed curve [24]. Finally, the age-adjusted PI–LL targets for each age group were determined based on two HRQOL outcome assessment tools.

A two-tailed *p* < 0.05 was considered significant. All analyses were performed using R version 4.3.1 for Windows.

## 3. Results

### 3.1. Demographics

Of the 280 patients enrolled in this study, 96 (34.3%) were male patients, and 184 (65.7%) were female patients, with a mean age of 64.7 ± 11.7 years. The mean BMI was 27.1 ± 5.7 kg/m^2^. A total of 196 patients (70.0%) had complications, and the mean CCI index was 4.5 ± 1.7. The length of stay was 10.9 ± 4.1 days. The posterior instrumentation used was pedicle screws in all patients, and they underwent 4.9 ± 2.2 levels of fusion. PI, PI–LL, LL, SVA, SRS-22 score, and ODI in the preoperative periods were 44.1 ± 10.7°, 34.5 ± 12.3°, 9.6 ± 5.7°, 5.5 ± 3.6 cm, 2.3 ± 1.1, and 31.1 ± 6.0, respectively. PI–LL, LL, and SVA in the postoperative periods were 15.3 ± 5.8°, 28.7 ± 14.0°, and 3.3 ± 2.6 cm, respectively. PI–LL, LL, SVA, SRS-22 score, and ODI at the time of the last follow-up were 20.7 ± 8.6°, 23.4 ± 14.1°, 4.0 ± 2.2 cm, 3.7 ± 0.8, and 18.3 ± 5.5, respectively (Table 1). Among all the covariates, osteopenia was partially missing in 56 cases (20.0%).

### 3.2. PI–LL and SRS-22 Score

In Group A, compared to the ++ grade PI–LL group, the 0 grade PI–LL group had a 1.0-fold increased probability of a good functional outcome (OR = 2.0, 95% CI 0.4–9.5, *p* = 0.383), while the + grade PI–LL group had a 0.3-fold increased probability of a good functional outcome (OR = 1.3, 95% CI 0.3–5.3, *p* = 0.656). In Groups B and C, the + grade PI–LL group seemed to have the best functional outcome (OR = 3.5, 95% CI 1.1–11.6, *p* = 0.035; OR = 2.3, 95% CI 0.1–34.8, *p* = 0.525), while the 0 grade PI–LL group was moderate in functional score (OR = 1.5, 95% CI 0.5–4.7, *p* = 0.469; OR = 1.4, 95% CI 0.2–7.6, *p* = 0.657). In Group D, compared to the ++ grade PI–LL group, the 0 grade PI–LL group had a 0.7-fold reduced probability of a good functional outcome (OR = 0.3, 95% CI 0.1–1.0, *p* = 0.050), while the + grade PI–LL group had a 0.4-fold reduced probability of a good functional outcome (OR = 0.6, 95% CI 0.2–1.2, *p* = 0.189) (Table 2).

**Figure 1:**
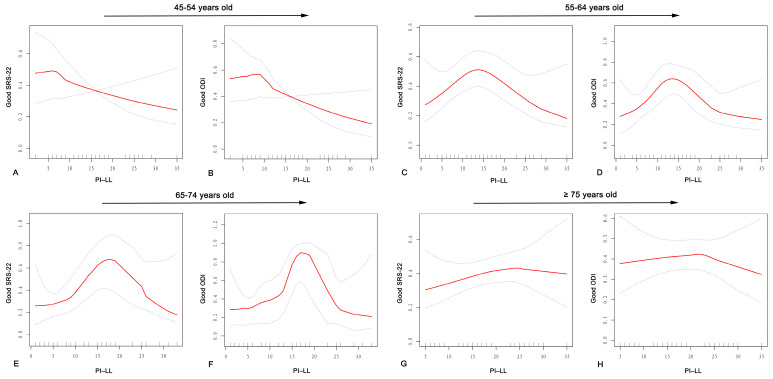
Curve-fitting plots of the PI–LL and HRQOL. The red line indicates the relationship between PI–LL and HRQOL, and the blue line indicates the 95% confidence interval. (**A**,**B**) show the relationship between PI–LL and HRQOL scores in the 45–54 age group; (**C**,**D**) show the relationship between PI–LL and HRQOL scores in the 55–64 age group; (**E**,**F**) show the relationship between PI–LL and HRQOL scores in the 65–74 age group; and (**G**,**H**) show the relationship between PI–LL and HRQOL scores in the ≥75 age group.

In the curve-fitting graphs, it could be seen that the relationship between PI–LL and the SRS-22 score was non-linear in each age group (Figure 1). In the further two-piecewise regression model, the position of the inflection point and the regression coefficient and confidence interval of the two-piecewise regression were clarified. In Group A, the calculated inflection point was 6°. To the left of this inflection point, the OR was 1.0 (95% CI 0.8–1.1, *p* = 0.889); to the right, the OR was 0.9 (95% CI 0.9–1.0, *p* = 0.102). In Group B, the inflection point was determined to be 14°. On the left, the OR was 1.2 (95% CI 1.0–1.4, *p* = 0.034), while on the right, the OR was 0.8 (95% CI 0.7–0.9, *p* = 0.041). For Group C, an inflection point was identified at 19°. To the left of the inflection point, the OR was 1.1 (95% CI 0.9–1.2, *p* = 0.262), and to the right, the OR was 0.9 (95% CI 0.6–1.3, *p* = 0.769). In Group D, the inflection point was 25°. To the left of this point, the OR was 1.1 (95% CI 0.9–1.2, *p* = 0.067), and to the right, the OR was 1.0 (95% CI 0.5–1.7, *p* = 0.925).

### 3.3. PI–LL and ODI Score

In Group A, compared to the ++ grade PI–LL group, the 0 grade PI–LL group had a 2.1-fold increased probability of a good functional outcome (OR = 3.1, 95% CI 0.6–14.8, *p* = 0.142), while the + grade PI–LL group had a 0.6-fold increased probability of a good functional outcome (OR = 1.6, 95% CI 0.4–5.9, *p* = 0.443). In Groups B and C, the + grade PI–LL group seemed to have the best functional outcome (OR = 2.4, 95% CI 1.1–5.5, *p* = 0.032; OR = 2.8, 95% CI 1.0–7.8, *p* = 0.050), while the 0 grade PI–LL group was moderate in functional score (OR = 1.8, 95% CI 0.7–4.0, *p* = 0.158; OR = 1.6, 95% CI 0.6–4.0, *p* = 0.289). In Group D, compared to the ++ grade PI–LL group, the 0 grade PI–LL group had a 0.6-fold reduced probability of a good functional outcome (OR = 0.4, 95% CI 0.1–1.5, *p* = 0.183), while the + grade PI–LL group had a 0.3-fold reduced probability of a good functional outcome (OR = 0.7, 95% CI 0.3–2.1, *p* = 0.629) (Table 3).

It can be found from the smoothed curves that the PI–LL had a non-linear relationship with the ODI score (Figure 1). On the left of the inflection point in Group A, the OR, 95% CI, and *p*-value were 1.1, 0.6 to 2.1, and 0.623, respectively. On the right of the inflection point, the OR, 95% CI, and *p*-value were 0.9, 0.8 to 1.0, and 0.200, respectively. In Group B, an inflection point was calculated to be 13°. On the left of the inflection point, the OR, 95% CI, and *p*-value were 1.3, 1.0 to 1.6, and 0.025, respectively. On the right of the inflection point, the OR, 95% CI, and *p*-value were 0.9, 0.8 to 1.0, and 0.107, respectively. In Group C, the inflection point was calculated to be 17°. On the left of the inflection point, the OR, 95% CI, and *p*-value were 1.2, 1.0 to 1.3, and 0.008, respectively. On the right of the inflection point, the OR, 95% CI, and *p*-value were 0.8, 0.5 to 1.2, and 0.342, respectively. In Group D, the inflection point was 23°. To the left of this point, the OR was 1.0 (95% CI 0.9–1.1, *p* = 0.847), and to the right, the OR was 0.9 (95% CI 0.4–1.6, *p* = 0.642) (Table 4).

### 3.4. PI–LL Targets Account for Age

Higher and lower thresholds for optimal surgical goals were determined using the two-piecewise regression model from the SRS-22 score and the ODI score. For each specific patient, the optimal PI–LL target needs to be determined according to the age group (Table 5).

## 4. Discussion

In spinal corrective surgery, increased attention has been paid to individual patient characteristics such as age and HRQOL. The aim of this study was to determine age-adjusted PI–LL targets for adult degenerative scoliosis. The current findings suggested that each age group had an age-specific PI–LL target. For patients aged 45–54 years, the operative realignment target was 6–9°; for patients aged 55–64 years, the operative realignment target was 13–14°; for patients aged 65–74 years, the operative realignment target was 17–19°; and for patients aged 75 years and older, the operative realignment target was 23–25°. It is worth noting that the conclusions of this study apply to the elderly Chinese population (over 45 years old) and may not be applicable to patients from other ethnic groups or physiological ages.

Patient-specific surgical planning and treatment are very important [25]. With age, the body undergoes physiological degenerative changes in the bones, muscles, ligaments, cartilage, and nervous system [7,10,11,26]. In the elderly population, the body’s overall alignment shifts forward, and the pelvis moves backward while maintaining the gravity line [27,28,29,30]. A recent large community-based cohort study of 1461 individuals for determining normal values of spinopelvic alignment showed that SVA, thoracic kyphosis, PI–LL, and pelvic tilt increased with age, and LL decreased with age [31]. These data suggest that preoperative planning for patients with ADS does not need to be fully implemented in accordance with the standards of a healthy population. Our results also showed that for patients older than 54 years, it was not necessary to meet the standard of PI–LL < 10°.

Park et al. stratified all patients into aligned or malaligned groups based on PI–LL > 10° and SVA > 50 mm. The data showed that in the younger cohort, postoperative ODI scores were significantly higher in the malaligned group than in the aligned group [32]. Unfortunately, however, they did not address specific preoperative planning strategies.

In the past, some authors have advocated overcorrection to mitigate loss of correction during follow-up since these patients were more likely to experience deterioration of overall balance due to degenerative changes over time [33,34]. However, accumulating evidence suggests that this practice may be harmful to elderly patients. In addition, overcorrection further increases the risk of mechanical complications, especially proximal junctional kyphosis (PJK) [35]. Byun et al. analyzed the impact of the LL correction on PJK in the context of age-adjusted sagittal balance goals. Results showed that overcorrection of LL relative to PI tended to increase the incidence of PJK after accounting for age-adjusted ideal sagittal alignment [36].

This study has several limitations. First, this study is a retrospective cohort analysis; thus, it is challenging to entirely eliminate bias. In the future, randomized controlled trials or prospective cohort studies with larger sample sizes should be conducted on this topic to verify the current findings. Second, the conclusions of this study were analyzed and verified in the Chinese elderly population and may not be suitable for Western countries. Due to differences in daily behavior and living habits, different races may have different optimal spinal and pelvic parameters. In addition, all patients in the cohort were older than 45 years, so the conclusions apply only to this population. Finally, in our study, we focused on chronological age as a primary factor due to the availability of robust data and its widespread use in clinical practice. However, we acknowledge that physical age (such as lower limb strength and walking speed) can significantly impact postoperative recovery and long-term health-related quality-of-life outcomes. Given the importance of physical age, future studies should incorporate assessments of physical fitness and reserve abilities. This could include objective measures (incorporating tests such as gait speed, timed up-and-go, and lower extremity strength assessments to provide a comprehensive evaluation of physical age); subjective measures (including patient-reported outcomes on physical function and activity levels); and integrated models (developing predictive models that combine chronological age, physical age indicators, and other relevant factors to better tailor surgical targets and improve outcomes).

## 5. Conclusions

This study showed that the optimal PI–LL after corrective surgery in ADS patients should be adjusted according to age. For older patients, previously used criteria may be at risk of overcorrection, which may result in lower HRQOL.

## Figures and Tables

**Table 1:** Summary of clinical and radiographic measurements of 280 patients undergoing corrective surgery. Values were expressed as number (%) or mean ± SD.

Variables	Overall (*N* = 280)
Age, years	64.7 ± 11.7
Sex	
Female	184 (65.7%)
Male	96 (34.3%)
BMI, kg/m^2^	27.1 ± 5.7
aCCI	4.5 ± 1.7
Osteopenia	
Yes	112 (40.0%)
No	112 (40.0%)
Missing	56 (20.0%)
Length of stay, days	10.9 ± 4.1
Estimated blood loss, ml	649.7 ± 447.4
Operative duration, min	281.7 ± 67.6
ASA score	2.2 ± 0.8
Number of fusion levels	4.9 ± 2.2
PI (°)	44.1 ± 10.7
Preoperative PI–LL (°)	34.5 ± 12.3
Postoperative PI–LL (°)	15.3 ± 8.3
PI–LL at follow-up (°)	20.7 ± 8.6
Preoperative LL (°)	9.6 ± 5.7
Postoperative LL (°)	28.7 ± 14.0
LL at follow-up (°)	23.4 ± 14.1
Preoperative SVA (cm)	5.5 ± 3.6
Postoperative SVA (cm)	3.3 ± 2.6
SVA at follow-up (cm)	4.0 ± 2.2
Preoperative SRS-22 score	2.3 ± 1.1
SRS-22 score at follow-up	3.7 ± 0.8
Preoperative ODI	31.1 ± 6.0
ODI at follow-up	18.3 ± 5.5

BMI, body mass index; aCCI, age-adjusted Charlson comorbidity index; ASA, American Society of Anesthesiologists; PI, pelvic incidence; PI–LL, pelvic incidence minus lumbar lordosis mismatch; LL, lumbar lordosis; SVA, sagittal vertical axis; SRS-22, Scoliosis Research Society-22; ODI, Oswestry Disability Index.

**Table 2:** Unadjusted and adjusted models for SRS-22 scores stratified by age. Values are odds ratio (OR) (95% CI).

Variable	Crude Model ^a^	Minimally Adjusted Model ^b^	Fully Adjusted Model ^c^
Group A (between 45 and 54 years old)			
0 grade PI–LL	2.5 (0.5–11.0), *p* = 0.204	2.2 (0.4–9.8), *p* = 0.320	2.0 (0.4–9.5), *p* = 0.383
+ grade PI–LL	1.0 (0.3–3.5), *p* = 0.965	1.1 (0.3–3.7), *p* = 0.917	1.3 (0.3–5.3), *p* = 0.656
++ grade PI–LL	Reference	Reference	Reference
Group B (between 55 and 64 years old)			
0 grade PI–LL	1.4 (0.4–4.5), *p* = 0.509	1.4 (0.4–4.7), *p* = 0.501	1.5 (0.5–4.7), *p* = 0.469
+ grade PI–LL	2.2 (0.7–7.0), *p* = 0.154	1.6 (0.4–5.2), *p* = 0.437	3.5 (1.1–11.6), *p* = 0.035
++ grade PI–LL	Reference	Reference	Reference
Group C (between 65 and 74 years old)			
0 grade PI–LL	1.9 (0.5–7.4), *p* = 0.320	2.2 (0.4–11.0), *p* = 0.322	1.4 (0.2–7.6), *p* = 0.657
+ grade PI–LL	2.1 (0.4–9.1), *p* = 0.323	2.8 (0.6–12.3), *p* = 0.155	2.3 (0.1–34.8), *p* = 0.525
++ grade PI–LL	Reference	Reference	Reference
Group D (age greater than 75 years)			
0 grade PI–LL	0.2 (0.1–0.9), *p* = 0.050	0.3 (0.1–1.1), *p* = 0.071	0.3 (0.1–1.0), *p* = 0.050
+ grade PI–LL	0.4 (0.1–1.2), *p* = 0.116	0.4 (0.1–1.1), *p* = 0.085	0.6 (0.2–1.2), *p* = 0.189
++ grade PI–LL	Reference	Reference	Reference

^a^ Crude model: we did not adjust other covariants. ^b^ Minimally adjusted model: we adjusted sex and age. ^c^ Fully adjusted model: we adjusted sex, age, BMI, and osteopenia. PI–LL, pelvic incidence minus lumbar lordosis mismatch; SRS-22, Scoliosis Research Society-22.

**Table 3:** Unadjusted and adjusted models for ODI scores stratified by age. Values are odds ratio (OR) (95% CI).

Variable	Crude Model ^a^	Minimally Adjusted Model ^b^	Fully Adjusted Model ^c^
Group A (between 45 and 54 years old)			
0 grade PI–LL	3.3 (0.7–14.5), *p* = 0.110	3.4 (0.7–15.0), *p* = 0.105	3.1 (0.6–14.8), *p* = 0.142
+ grade PI–LL	1.7 (0.5–6.3), *p* = 0.364	1.7 (0.4–6.1), *p* = 0.404	1.6 (0.4–5.9), *p* = 0.443
++ grade PI–LL	Reference	Reference	Reference
Group B (between 55 and 64 years old)			
0 grade PI–LL	1.1 (0.3–3.6), *p* = 0.855	1.2 (0.3–3.8), *p* = 0.779	1.8 (0.7–4.0), *p* = 0.158
+ grade PI–LL	1.2 (0.4–4.0), *p* = 0.686	1.6 (0.5–5.3), *p* = 0.429	2.4 (1.1–5.5), *p* = 0.032
++ grade PI–LL	Reference	Reference	Reference
Group C (between 65 and 74 years old)			
0 grade PI–LL	1.3 (1.0–1.7), *p* = 0.043	1.1 (0.9–1.4), *p* = 0.095	1.6 (0.6–4.0), *p* = 0.289
+ grade PI–LL	2.3 (0.3–14.5), *p* = 0.372	2.5 (0.6–10.6), *p* = 0.210	2.8 (1.0–7.8), *p* = 0.050
++ grade PI–LL	Reference	Reference	Reference
Group D (age greater than 75 years)			
0 grade PI–LL	0.7 (0.2–2.9), *p* = 0.711	0.7 (0.2–3.0), *p* = 0.740	0.4 (0.1–1.5), *p* = 0.183
+ grade PI–LL	1.0 (0.3–2.8), *p* = 0.944	0.9 (0.3–2.6), *p* = 0.878	0.7 (0.3–2.1), *p* = 0.629
++ grade PI–LL	Reference	Reference	Reference

^a^ Crude model: we did not adjust other covariants. ^b^ Minimally adjusted model: we adjusted sex and age. ^c^ Fully adjusted model: we adjusted sex, age, BMI, and osteopenia. PI–LL, pelvic incidence minus lumbar lordosis mismatch; ODI, Oswestry Disability Index.

**Table 4:** Results of two-piecewise regression model. The inflection point was set according to the PI–LL at the peak of the curve.

Age Group	SRS-22 Scores			ODI Scores		
	Inflection Point	OR (95% CI)	*p*-Value	Inflection Point	OR (95% CI)	*p*-Value
Group A (between 45 and 54 years old)	≤6°	1.0 (0.8–1.1),	*p* = 0.889	≤9°	1.1 (0.6–2.1),	*p* = 0.623
	>6°	0.9 (0.9–1.0),	*p* = 0.102	>9°	0.9 (0.8–1.0),	*p* = 0.200
Group B (between 55 and 64 years old)	≤14°	1.2 (1.0–1.4),	*p* = 0.034	≤13°	1.3 (1.0–1.6),	*p* = 0.025
	>14°	0.8 (0.7–0.9),	*p* = 0.041	>13°	0.9 (0.8–1.0),	*p* = 0.107
Group C (between 65 and 74 years old)	≤19°	1.1 (0.9–1.2),	*p* = 0.262	≤17°	1.2 (1.0–1.3),	*p* = 0.008
	>19°	0.9 (0.6–1.3),	*p* = 0.769	>17°	0.8 (0.5–1.2),	*p* = 0.342
Group D (age greater than 75 years)	≤25°	1.1 (0.9–1.2),	*p* = 0.067	≤23°	1.0 (0.9–1.1),	*p* = 0.847
	>25°	1.0 (0.5–1.7),	*p* = 0.925	>23°	0.9 (0.4–1.6),	*p* = 0.642

Adjusted for sex, age, BMI, and osteopenia. PI–LL, pelvic incidence minus lumbar lordosis mismatch; SRS-22, Scoliosis Research Society-22; ODI, Oswestry Disability Index.

**Table 5:** Age-adjusted PI–LL targets for each age group were determined based on HRQOL outcome assessment tools.

Operative Realignment Targets	Age Group, Years			
	45–54	55–64	65–74	≥75
Lower threshold	6°	13°	17°	23°
Higher threshold	9°	14°	19°	25°

PI–LL, pelvic incidence minus lumbar lordosis mismatch; HRQOL, health-related quality of life.

## Data Availability

The datasets used and/or analyzed during this study are available from the corresponding author upon reasonable request.
